# Upcycled Foods: What Influences Consumer Responses to a Circular Economy-Based Consumption Strategy? Insights from a Systematic Literature Review

**DOI:** 10.3390/foods15020364

**Published:** 2026-01-20

**Authors:** Qamar U Zaman, Luca Rossetto, Leonardo Cei

**Affiliations:** Department of Land Environment, Agriculture and Forestry, University of Padova, 35122 Padova, Italy; luca.rossetto@unipd.it (L.R.); leonardo.cei@unipd.it (L.C.)

**Keywords:** upcycled foods, food waste, circular economy, consumer preferences, consumption

## Abstract

Upcycled foods (UFs) are foods that are produced from ingredients that would otherwise be wasted and are considered a sustainable solution to the issue of food waste. However, since consumers’ responses to these foods will ultimately determine their success, there is a need to identify the factors that affect such responses. This systematic review is intended to contribute to fulfilling this need. A literature search was conducted in Scopus on 10 July 2025. Following the PRISMA protocol and setting selected inclusion criteria (scientific papers on consumer evaluation of UFs published since 2010 in English), 54 research articles (83 studies) were analyzed. The findings are discussed through the lens of the Total Food Quality model, where product cues, combined with consumers’ characteristics and perceptions, develop consumers’ ultimate responses, such as general attitude (analyzed in 91.7% of the reviewed studies), purchase intention (77.4%), sensory evaluation (69.2%), and willingness to pay (66.7%). Despite the general positive consumer attitudes toward UFs, translation into actual purchasing behavior is not immediate, and consumer awareness appears to be a major obstacle. However, the analysis of the literature suggests promising strategies to widen the acceptance and consumption of UFs. These entail the use, for example, of informational tools (e.g., claims and certifications), which can be differentiated to target consumers with different levels of knowledge and appreciation of UFs. In addition, targeting specific consumer segments (e.g., environmentalists) can promote a faster acceptance and spread of UFs, while providing information about the nature of UFs will likely help to reduce relevant barriers, such as price sensitivity, risk aversion, and food and technology neophobia.

## 1. Introduction

Sustainability in food production and consumption, as originally defined, refers to a systemic approach that meets current environmental needs without compromising the ability of food processes to complete their natural cycle [1,2]. This concept holds significant importance in the food and agricultural sector, particularly concerning the United Nations’ Sustainable Development Goals (SDGs) [3,4,5]. SDG2 aims to achieve zero hunger by 2030, while SDG12 promotes sustainable production and consumption patterns [6]. Moreover, ensuring food security, defined as the provision of sustainable and nutritious diets to individuals and communities, remains a critical challenge of our time [7].

The anticipated challenges in delivering sustainable, healthy, and sufficient food are expected to intensify as the global population is projected to reach approximately 9 billion by 2050 [8]. From this perspective, food waste represents a significant global challenge for sustainable food production and consumption. Approximately one-third of the total food produced for human consumption is wasted or lost annually, leading to adverse consequences such as food insecurity, financial losses, and environmental degradation [9,10,11,12].

The annual global estimate of food waste is around 1.3 billion tons, occurring at various stages in the food supply chain, including production, storage, processing, distribution, retail, food service, and final consumption [13,14,15,16]. In addition to entail a cost of approximately USD 990 billion annually [17], this waste also contributes to 8–10% global greenhouse gas emissions [18]. In Europe, according to Eurostat, around 59 million tons of food waste are generated every year, with the highest share occurring at the household level (around 32 million tons) [19]. Therefore, mitigating food loss and waste stands out as a pivotal objective for attaining a sustainable food system [20].

To address this challenge, the valorization of food by-products has emerged as a potentially valuable approach, focusing on reusing, recycling, or upcycling food by-products that would otherwise be wasted during food manufacturing and processing [21,22,23]. Food waste valorization has been shown to produce positive environmental, economic, and social advantages [24].

The practices of food by-product valorization can be included under the broader umbrella of the circular economy. This concept is meant to be an alternative to the classic linear model characterized by the consecutive chain of production–consumption–disposal. By introducing concepts such as reusing, repairing, recycling, or reprocessing, by-product materials and leftover products can re-enter the production cycle [25].

The adoption of these concepts led to the implementation of several food production processes that can use a wide variety of food wastes, some examples being brewery or coffee spent grains [26,27], hazelnut residues [28], olive oil pomace [29], or by-products of the wine industry [30,31,32]. Foods that incorporate food residues and by-products as ingredients are called upcycled foods (UFs). Specifically, the Upcycled Food Association gives the following definition of UFs: “Upcycled foods use ingredients that otherwise would not have gone to human consumption, are procured and produced using verifiable supply chains, and have a positive impact on the environment” [33] (p. 2).

As a result, multiple upcycled products for human consumption have appeared, from biscuits made with grape pomace [34] to muffins made with banana residues [26] or upcycled chicken nuggets and ice creams [35].

However, a relevant question is how these products will perform on the market. Here, consumers play a major role, and their acceptance of these innovative products is crucial for the establishment of a relevant UF market. Specifically, two questions are of particular interest:

- What is the response of consumers toward these products?

Understanding whether consumers have a favorable opinion of this novel food category and whether they will introduce UFs into their diets will allow us to gauge the current potential of the UF market.

b. Which factors shape consumers’ opinions and decisions about UFs?

Answering this question will help identify possible ways to promote and spread their consumption as a means to lower the environmental impact of food chains.

These two questions constitute the research questions of our work. As the literature has already extensively investigated, albeit at different levels, the relationship that consumers have with UF products, a systematic literature review appears to be the best strategy to summarize the scattered evidence collected by other authors and attempt to condense them to answer our two research questions. In this respect, some efforts have already been made in this direction. Swaraj et al. [36], for example, in reviewing potential barriers to the development of the UF sector, identify some consumer elements that may hinder such development. In this respect, our exclusive focus on consumers allows for a more thorough investigation of the relationship of consumers with UFs, not just through a “barrier” perspective, but also by analyzing factors potentially prompting positive responses. A similar approach was adopted by Moshtaghian et al. [37] (who, however, did not adopt a systematic approach) and Aschemann-Witzel and Stangherlin [2] in their reviews on UF consumption. Our contribution adds to these works in two ways. First, we extend the period of analysis, which, in a rapidly growing literature sector, allows us to add a relevant amount of new evidence. Second, in contrast to their ex-post, inductive syntheses of reviewed studies, we synthesize the literature using a pre-determined theoretical framework. This allows us to better connect the collected evidence to consumption theory, thus possibly deriving more insightful conclusions.

Indeed, to guide our inquiry, we ground the review in the Total Food Quality (TFQ) model [38], a framework often used in consumer studies to describe the formation of consumers’ intentions. The choice of this theoretical framework, which is explained in detail in Section 3, was guided by its comprehensiveness. Most consumer acceptance frameworks tend to place their main focus either on product characteristics (e.g., cue utilization theory [39], Lancaster demand theory [40]) or on consumer characteristics (as in behavioral models of consumption, such as the Theory of Planned Behavior [41] or the Norm Activation Model [42]). Conversely, the TFQ model considers both product attributes as well as their perception by consumers, thus recognizing the role played by both product and consumer characteristics in product evaluation. In addition, the TFQ model takes into account both ‘before purchase’ and ‘after purchase’ factors that might affect consumer evaluations, with the aim of providing a more comprehensive overview of the consumption dynamics. In our view, such a level of comprehensiveness allows us to more thoroughly synthesize the evidence obtained from the literature.

The methodology used to conduct the systematic review is illustrated in the next section. In the following section, we report the results. Finally, a discussion and a conclusive section summarize the main evidence collected from the revised literature, offering relevant reflections and potential suggestions for the future development of the UF sector, at least with respect to demand-related aspects.

## 2. Materials and Methods

To address our research questions, we conducted a systematic literature review. Following Stiletto et al. [43], we integrated the three-step approach proposed by Tranfield et al. [44]—planning, conducting, and reporting the review—with the Preferred Reporting Items for Systematic Reviews and Meta-Analysis (PRISMA) reporting guidelines from Page et al. [45] (no protocol was registered). The first step, planning the review, involved clearly defining our research questions:

**RQ1:**  *What is the response of consumers toward UF products?*

**RQ2:**  *Which factors affect the response of consumers toward UF products?*

In this context, we define “response” as a broad spectrum of consumer reactions, which range from consumer perceptions to actual purchasing behaviors. This definition, as illustrated later, served to clearly define one of the eligibility criteria to be used in the retrieved studies.

This comprehensive approach aligns with the scope of consumer economics, which extends beyond final purchasing patterns to include the analysis of preferences and motivations underlying consumption choices. Furthermore, this broad definition allows us to examine whether UFs elicit different responses depending on the type of evaluation, potentially revealing that positive perceptions do not necessarily translate into positive purchasing behaviors.

Our target population of studies included those evaluating consumer responses to UF products. We limited our inquiry to research listed in the Scopus bibliographic database. Although this must be considered a limitation of our study, Scopus has been recognized as being wider in scientific coverage than other bibliographic databases [46]. In addition, the usually mentioned limitations of Scopus, like worse coverage of old research [47] and limited availability of books and conference proceedings [48], were not relevant for this task. Indeed, to ensure the relevance and quality of our review, we applied the following inclusion and exclusion criteria:

- Only peer-reviewed scientific articles were included to ensure robust, validated results.
- Articles were limited to those written in English, focusing on internationally accessible research.
- Publications before 2010 were excluded to focus on recent evidence, as earlier material on this topic is scarce.

The second step of the approach by Tranfield et al. [44] in conducting their review involved creating and implementing a search strategy. Based on our research questions, we developed the following search string:

TITLE-ABS-KEY (food AND consumer AND (“circular economy” OR upcycl* OR recycl* OR reprocess* OR “waste to value” OR “value added surplus” OR “rescue based”))

To keep the initial search wide enough not to miss relevant research, we included, in the search string, terms that are sometimes used as synonyms of “upcycled” (“reprocessed”, “recycled”, “rescued”) [49]. In addition, some terms are used in the literature to indicate specific concepts that, given the broad scope of the definition of UFs (see Section 1), can be grouped with “upcycled”. That was the case for “waste-to-value foods”, which entails the “reuse of waste and by-products to increase the nutritional power of food or its shelf-life extension” [50] (p. 2), and “value-added surplus products”, which are defined as “foods made from surplus ingredients that would have been otherwise wasted” [49] (p. 57).

**Figure 1:**
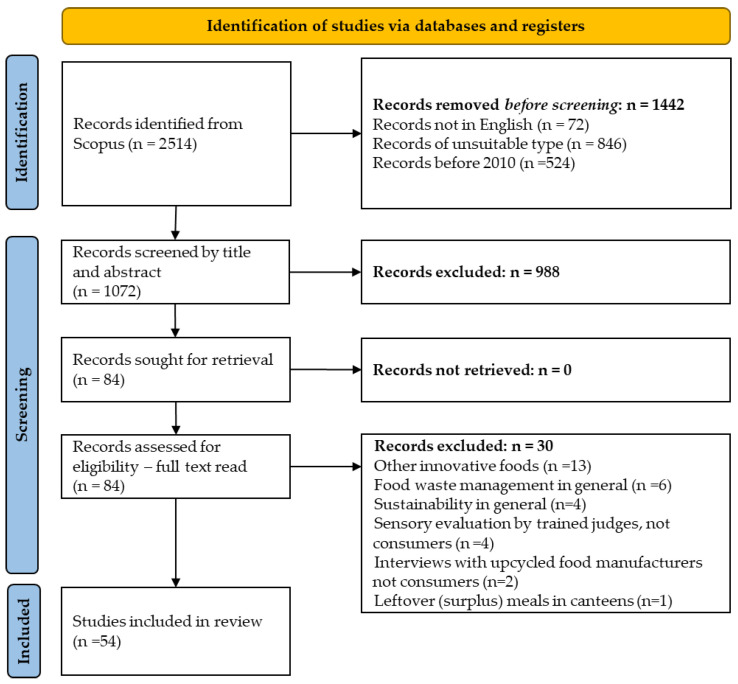
Literature retrieval and selection based on the PRISMA flow diagram.

This initial search, conducted on 10 July 2025, provided 2514 bibliographic entries. Applying the filters corresponding to our inclusion and exclusion criteria (document type = “Article”; language = “English”; Year = 2010–2023) returned 1442 documents. As per the PRISMA flow diagram (Figure 1), this constitutes the identification phase of our systematic review process.

The screening phase involved a systematic evaluation of bibliographic entries by one of the researchers, conducted in three consecutive stages: initial screening based on titles, followed by abstract review, and concluding with full-text assessment.

The following screening criteria were used:

- The products considered are UFs, according to the definition provided by the Upcycled Food Association (see Section 1).
- The study deals with consumers, with no restriction on consumer type. Studies involving only expert panel evaluation were excluded.
- The study investigates consumer responses to UFs, where “responses” can include attitudes and perceptions, acceptability, purchase intention and willingness to buy (WTB), willingness to pay (WTP), sensory evaluation, and actual purchase. Papers investigating multiple responses were split into separate studies.
- All study designs were eligible, while purely methodological works were discarded.

The screening process resulted in the selection of 54 articles for inclusion in our review.

The third step involves reporting the results. In this section, we present the bibliometric characteristics of the included material, while a detailed analysis of the content follows in the subsequent section of the manuscript. Specifically, the analysis of the content required the collection of information from each included study on two classes of elements: (i) consumer response to UFs; (ii) factors that affect consumer responses. For both classes, a vote-counting approach by direction of the effect was adopted. For the first class, we counted the number of studies with different consumer response directions (negative, null, or positive). For the second class, we counted, for each analyzed factor, the number of studies with different effect directions of the factor on the consumer response. The factors in the latter class were further categorized into (i) product cues; (ii) consumer characteristics; (iii) consumer perceptions; and (iv) experienced quality. This classification, as explained in Section 3, was based on a theoretical model of consumer behavior (the Total Food Quality model), which served as a framework to synthesize and report the review results. The collection of data from the reviewed studies was initially performed by one of the authors, while a validation of all analyzed records was performed by a different one.

**Figure 2:**
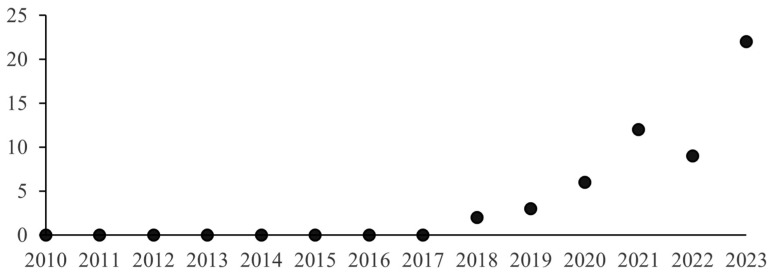
Number of publications per year (2010–2023).

A temporal analysis of the selected literature (Figure 2) reveals that, within our defined time period, no paper was published before 2018. This observation underscores that the study of consumer preferences for UFs is an emerging field of research. Despite its nascent status, the topic has garnered significant attention in recent years, as evidenced by a notable increase in publications, particularly in the last few years.

**Figure 3:**
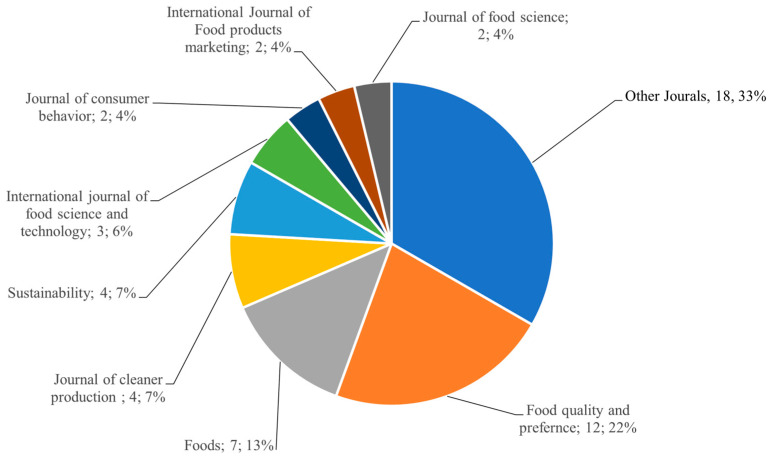
Number of papers published in different scientific journals.

Analysis of publication venues (Figure 3) reveals that *Food Quality and Preference* is the leading journal in publishing studies on consumer evaluations of UF products. However, beyond this primary source, the publication landscape appears notably diverse, with articles on the topic dispersed across a wide array of journals. This fragmentation might be attributed to the absence of specialized journals dedicated to UF products or to the lack of established research strands within existing journals.

**Figure 4:**
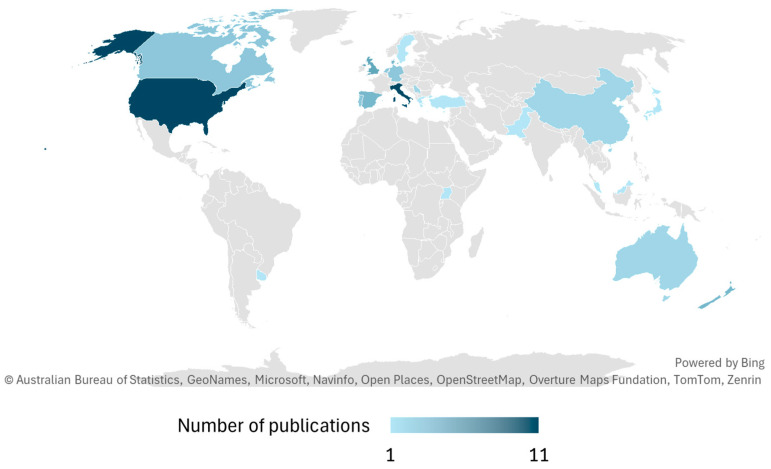
Number of articles published in different countries.

Finally, an analysis of the geographical distribution of the articles (Figure 4) reveals significant disparities across different regions. European countries dominate the landscape, followed by North America and Oceania. In contrast, Latin America and Africa are notably underrepresented in the literature. At the country level, Italy and the US display the highest number of studies (11 articles), followed by the UK (5 articles) and Canada, New Zealand, and Denmark (4 articles each).

## 3. Results

**Figure 5:**
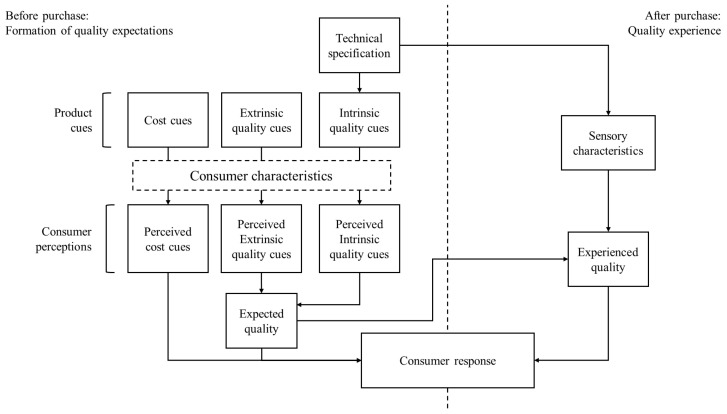
Modified version of the Total Food Quality model used as a theoretical framework.

To synthesize the retrieved literature, we employed an adapted version of the Total Food Quality model proposed by Grunert et al. [38] (Figure 5). Specifically, we kept the original two-dimensional structure. The horizontal dimension distinguishes between before-purchase expectations and after-purchase experience. The vertical dimension addresses the inference-making process through which consumers utilize a set of signals to form expectations (before purchase) or evaluations (after purchase) of product quality, ultimately leading to a specific response.

Most of the constructs of the original model, as well as the connections between them, were also maintained. However, we decided to remove four constructs and merge two others. With respect to the latter, while the original TFQ model distinguishes between a before-purchase and an after-purchase type of response (respectively, *intention to buy* and *future purchases*), we merged the two into a *consumer response* construct. This allowed us, on the one hand, to accommodate a larger set of consumer responses not limited to purchase intentions, in line with the objectives of the review. On the other hand, in scientific studies, *experienced quality* is usually operationalized, especially for novel product categories, using experimental tastings. This means that what is commonly measured is still often a pre-purchase response, as consumers never really bought the product.

With respect to removed constructs, two of them relate to the pre-purchase stage. Here, we dropped the *perceived costs* (which in the original model mediate the relation between the *perceived cost cues* and the *intention to buy*) and the *expected purchase motive fulfillment* constructs (which is the mediator of a further indirect effect of *expected quality* on *intention to buy*). In the after-purchase stage, we removed *meal preparation* and *experienced purchase motive fulfillment*. The former, which represents the way in which consumers decide to prepare food at home, is affected by the *perceived intrinsic quality cues* and the *expected quality*, and in turn affects the *experienced quality*. The latter plays a similar role in the *expected purchase motive fulfillment*, mediating the effect of *expected quality* on *future purchases*.

The decision to remove the four constructs was made to keep the model simple, in light of the objectives of the review. *Perceived costs* could be, in fact, conveniently integrated into the *perceived cost cues*, at least for the costs directly connected to UFs, which were the ones of interest. *Meal preparation* is a construct of a rather technical nature, which falls beyond the scope of this review. *Purchase motive fulfillment* (*expected* and *experienced*), in addition to not being investigated for UFs by researchers (as ex-post evidence), was likely related to more general consumption motives, and not intimately related to UF.

Finally, we added to the original model an additional construct, *consumer characteristics*, as a moderator between the *product cues* category and the *consumer perceptions* category. Indeed, several behavioral models, e.g., the Theory of Reasoned Actions [51], Norm Activation Model [42], and Theory of Planned Behavior [41], posit that an individual’s perception of a product is significantly influenced by their personal characteristics, including socio-demographics, attitudes, interests, and intrinsic values.

The presentation of the results below is therefore arranged according to four classes, in line with the revised TFQ model structure: *product cues*, *consumer characteristics*, *consumer perceptions*, and *sensory characteristics*. The coding used is reported in Table A1 in Appendix A.

### 3.1. Consumer Responses

While the original TFQ model focuses on the intention to purchase (or repurchase) a specific food product, our review adopts a broader perspective, considering a more comprehensive *consumer response* category. This decision was taken considering that purchase intention is just one of several ways to investigate consumer preferences for food products, which can be assessed through multiple indicators. Before delving into the effect of various factors on consumer responses, it is crucial to understand how the literature evaluates consumer preferences for UF products and what the outcomes of these assessments are.

The main indicators used to assess consumer responses towards UFs are purchase intention or WTB (37.35%), consumer acceptability (18.07%), WTP and general perceptions (14.46% each), and sensory evaluation (15.66%) (Table 1). Purchase intention or WTB is basically the intention to purchase or buy UFs. Consumer acceptability is the degree to which consumers are open to accepting, trying, and including UFs in their diets. WTP is the intention or willingness of consumers to pay a price premium for UFs. General perceptions are the attitudes that consumers have about UFs, which may not necessarily translate into actual intention or behavior. Finally, sensory evaluation includes the actual experience of UFs based on sensory parameters such as smell, aroma, texture, and taste.

Most studies (77.11%) reveal a positive inclination of consumers towards UF products, which is particularly evident when examining general perceptions [10,49,52,53]. Consumers generally view these products as environmentally beneficial, particularly in reducing food waste [54,55]. Additional positive perceptions include improved taste [54] and enhanced nutritional and food safety characteristics [10].

These positive perceptions often translate into high acceptability of UFs across diverse contexts, from olive oil by-products [56] to bread enriched with wine by-products [57], seafood by-products [58], or upcycled ingredients soups [59]. Similarly, consumers appear willing to try these products, as 77.42% of the studies identified positive WTB and purchase intentions [50,60,61,62]. In some instances, purchase intention can be enhanced by specific communication strategies, such as providing rational messages [63], appropriately designed logos [64], or clearly listing the product ingredients [26]. However, some studies found that information and communication strategies are essential to stimulate purchase intention, which would otherwise be absent among consumers [65,66]. Indeed, in certain cases, as observed in New Zealand [67] and Spain [68], the presence of upcycled ingredients actually reduces the acceptability of food products.

Even more heterogeneous results are observed when investigating WTP. In this case, two-thirds of the studies found that consumers are willing to pay a price premium for UFs, primarily as a recognition of their environmental benefits [35,57,69]. However, a significant proportion of studies estimated negative price premiums, sometimes attributed by the authors to inadequate communication of the positive aspects of UFs [35] or consumer price sensitivity [70].

What emerges is a pattern where positive consumer responses, although prevalent, decrease as consumers approach actual consumption. When asked about their general attitudes, almost all studies agree on a positive perception of UF. However, when consumers are questioned about potential actual behavior (such as intention to buy or pay), heterogeneity emerges. This evidence is not unexpected, as multiple factors can influence the path from attitudes to actual behavior, as theorized in several consumption behavior models. Additionally, multiple studies have empirically observed that, especially for sustainable food products, a significant gap exists both between consumers’ attitudes and intentions, as well as between intentions and behavior [71,72].

A final response investigated in the literature is product sensory evaluation. Here too, heterogeneity is relatively high, with 69.23% of the studies identifying positive results and 30.77% observing negative evaluations of UFs. In this regard, Costantini et al. [73] reported a higher overall liking for biscuits containing hazelnut skin, and similar results were observed for upcycled rice crackers [74], beef with upcycled ingredients [68], and muffins made from brewery spent grains [69]. Positive overall sensory evaluations were also noted by [75,76,77]. Conversely, consumers gave low scores to the sensory evaluation of cereal bars made with brewers’ spent grains [78] and negatively evaluated several sensory characteristics (e.g., taste, texture, and appearance) of upcycled burgers or bread made with tomato by-products [79,80].

### 3.2. Product Cues

According to the *cue utilization theory,* consumers use effective cues to make decisions [63,64,81]. In the TFQ model, *product cues* are divided into intrinsic, extrinsic, and cost cues. Intrinsic cues relate to the physical properties of the product, directly derived from its production method (*technical specification* in Figure 5), such as color, fat cover (e.g., for meat), or visible damage (e.g., for fruit). All other cues are considered extrinsic [39]. The TFQ model specifically separates cost cues, which primarily relate to price and other costs associated with obtaining the product (e.g., travel and time).

In the literature on UFs, cost cues are primarily investigated in terms of price, with several articles analyzing how the price of UFs affects consumers’ acceptance and preferences. Although in some contexts and for some consumer segments, it is not uncommon for price to act as a quality signal, the reviewed studies on UFs report the usual inverse relationship between price and measures of consumers’ responses [55,70,82]. For example, Ref. [26] observed that 56% of New Zealand consumers found UFs with brewers’ spent grains more attractive when priced lower. Similarly, in the US, Ref. [62] showed that consumers were willing to buy by-products of fruits, vegetables, seed oils, spent grains, cereals, fish, and meat if they had a lower price. According to Stelick et al. [78], the lower optimal price point of upcycled cereal bars with brewers’ spent grains was the main driving factor of their acceptability.

Interestingly, a study by Peschel et al. [83] suggests that cost transparency (i.e., acknowledging the additional costs required to produce upcycled products) can improve perceived price fairness, thus stimulating the acceptance of upcycled products.

The second type of cues we consider are extrinsic product cues. In the literature on UFs, these cues are mainly related to delivering information about desirable product characteristics, such as reduced environmental impact, beneficial nutritional and food safety effects, or their role in food waste management and the circular economy.

Regarding food safety, Moshtaghian et al. [10] observed that food safety information, for example, through traceability, contributes to consumer acceptability of UFs in Sweden. In Korea, Jeon [53] observed that food safety was the main reason for selecting UFs by consumers. Similarly, in the US, the presence of safety information about seafood by-products led to higher consumer acceptability [58].

A more important extrinsic cue, however, is environmental information, which positively affects consumers’ response to UFs. This is observed, for example, on the attitudes of European consumers by Aschemann-Witzel et al. [84]. Similarly, Goodman-Smith et al. [54] observed that most New Zealand consumers deem upcycled beer to produce more environmental benefits, be more sustainable, and have a lower carbon footprint. This, as shown by McCarthy et al. [61] in Australia, may lead respondents to display a willingness to buy UFs due to their perceived positive impact on the environment, or to turn perceived environmental sustainability into a WTP driver [82]. Similarly, in the US and China, consumers were more likely to try UFs as they perceived them as better for the environment [62].

Waste management is closely related to environmental. Indeed, adopting a ‘recycling’ perspective, the consumption of UF holds the promise of reducing food waste. Informing consumers about the nature of these foods may have positive effects on purchase behaviors. For example, Ref. [66] found that 89% of respondents were willing to buy UFs due to their association with reduced food waste, which was the highest predictor of purchase intention. Similar evidence has been obtained by Kawata and Kubota [85] and McCarthy et al. [86], where respondents’ willingness to buy UFs was strongly related to their perception that the food contributed to food waste reduction. However, in some cases, knowledge of the link between UF and food waste might be counterproductive. This was observed, for example, by de Visser-Amundson et al. [59], who highlighted that explicitly associating upcycled products with food waste might reduce purchase intention or lower the WTP due to risk-averse individuals relating food to waste [87].

This potential concern opens the way to another set of cues, namely health-related ones. Information about health attributes and nutritional value can thus enhance consumers’ purchase intention [65,88], their WTP [57,69,87] and their attitudes in general [62,89]. In part, this might be due to the attention to the presence of health attributes on food by some consumer segments. However, some consumers were also observed to perceive UFs as intrinsically healthier than their conventional counterparts [68,90], thus possibly reinforcing the effect of health cues on UFs. It is important to note that the reviewed literature does not investigate the objective nutritional characteristics of UFs, whose analyses lie within the scope of other research fields.

While information about the potential benefits of UFs can be conveyed through claims, official logos and certifications are alternative information tools that can be exploited in the market. In some cases, logos can be related to other certification schemes, which upcycled products might comply with. Ref. [63] observed that the presence of an organic label increased Chinese consumers’ purchase intention for upcycled chicken nuggets. Similarly, using the Carbon logo can improve consumers’ WTB [82] and WTP [69] for different types of UFs. However, specific logos indicating the upcycled nature of the products can be used. These logos seem to have a certain appeal among consumers. Bhatt et al. [49], for example, observed that an “upcycled” label is preferred over alternative labels indicating food waste reuse. Upcycled logos have been observed to increase the acceptance of UFs [26,49], as well as the intention to purchase or willingness to try food with upcycled ingredients [62,64].

Another piece of information that can affect the acceptability of UFs is the indication of ingredients. On the one hand, the indication of ingredients per se may improve the overall acceptability of the product, as observed in the case of upcycled meat [68]. Factors like ingredient origin were observed to be important elements contributing to the likeness of waste-to-value food products [50,65]. Additionally, specific ingredients used may increase consumer acceptance because they are associated with desirable characteristics, such as plant-based protein sources [82] and nutrient-rich seed oils (pumpkin and melons) bearing a geographical indication [80], communicating the naturalness of the product [57] or their origin [56].

The last type of product cues that might be relevant in the formation of consumer responses are intrinsic cues. Factors like color, visual appearance, or damage can influence consumers’ responses [49]. For example, Fernandez-Pan et al. [79] observed that the addition of tomato powder to flatbread may cause an intense coloration that is not appreciated by consumers. However, as intrinsic cues are highly product- and process-dependent, their role in determining the consumer response to UFs does not seem to be different from what is observed for conventional foods, and heterogeneity can be observed in the appreciation of intrinsic cues within the upcycled category [91]. As in the case of sensory characteristics (Section 3.5), however, consumer evaluation of intrinsic cues opens the way to the purposeful use of upcycled ingredients to modify specific intrinsic attributes of the product.

### 3.3. Consumer Characteristics

A significant portion of the literature on the acceptance of UFs has investigated consumers’ characteristics that can stimulate favorable intentions and behaviors toward these products. Among these characteristics, factors related to consumers’ familiarity with UFs and their knowledge play a major role. Familiarity is usually assessed by asking respondents whether they had already heard of UFs [26,50,66,82] or to specifically indicate their degree of familiarity with them [35,92]. Knowledge is investigated in subjective terms, asking respondents to self-evaluate their level of knowledge [50,52,57,82].

While most studies observe a low degree of familiarity with and knowledge of UFs, only a few of them investigate the effects of these consumer characteristics on consumer responses. Indeed, most studies use the experimental provision of information to operationalize consumer knowledge. In general, familiarity seems not to play a major role in stimulating favorable consumer responses [35,66]. Conversely, the role of knowledge seems more relevant, as it has been found to positively affect respondents’ acceptance of UFs [52,57] and their willingness to pay premium prices [92]. The role played by consumers’ previous knowledge of upcycled products thus reinforces the importance of informing consumers [64], for example, through extrinsic product cues, as highlighted in the previous section.

Socio-demographics and lifestyle-related aspects also constitute relevant consumer characteristics. However, as in studies on organic foods [93], the role of socio-demographic characteristics is not straightforward, and studies tend to provide heterogeneous evidence (see Table A2 in Appendix A for a summary of the effects of sociodemographic characteristics). In terms of gender, females tend to express more favorable responses and responses than males [84,94], but inconclusive results are observed as well [60,88,95]. The effect of education level is even less clear, with studies observing positive [60], null [84], and negative results [65]. Age-related findings are more homogeneous, with middle-aged individuals appearing to be more open to UFs according to [10]. However, discordant results are also observed in this case, such as in Hellali et al. [87], where young consumers showed higher WTP levels, or in Cattaneo et al. [95], where age wass not a discriminating factor in attitudes toward UF.

Generational levels of consumers have been found to have varying acceptability of UFs. Zhang et al. [96] found that baby boomers (born between 1944 and 1964) had the highest purchase intention for UFs among all generations. Conversely, Perito et al. [56] found that generations X (ages between 40 and 54) and Y (ages between 25 and 39) had the highest willingness to try UFs.

The heterogeneous evidence about socio-demographic characteristics can be explained if we consider these characteristics not as the primary drivers of consumers’ responses, but rather as proxies for broader social conditions, such as individual norms and values [97]. In this sense, consumers’ psychological and personality traits may be better explanatory factors for their preferences toward UFs.

In terms of personality, several traits have been investigated in the literature. Consumers’ environmental concerns are generally associated with more favorable attitudes and intention to buy UFs [61,82]. This ecological purchase behavior [98] aligns with the environmental benefits associated with these products. The positive image associated with UFs may also explain the positive attitudes and WTB observed in consumers looking for self-rewards [99], social desirability [84], and social status [61]. Although UFs are not necessarily linked to specific health benefits, some authors found that consumers who pay more attention to health in their purchasing behaviors display higher WTB and WTP values than other consumer groups [57].

Conversely, some personality traits can hinder the development of favorable responses toward upcycled products. Miolla et al. [57] observed that traditionalist consumers, who attach high importance to their traditional food habits and local food origin, are more reluctant to move toward innovative products, exhibiting lower WTB and WTP. Similarly, consumers’ price sensitivity can act as a barrier to the consumption of UFs [70]. In this case, the obstacle is represented not by an a priori aversion to this type of product, but instead by a reluctance to pay higher prices [70]. However, simple price consciousness does not necessarily act as a constraining factor, as highlighted in [61]. If price-conscious consumers perceive UFs to be worth the higher price, they might still be willing to spend more for them.

Finally, some psychological aspects related to the acceptance of UFs are found in the literature. Among these, food neophobia and food technology neophobia are prominent, representing reluctance towards or barriers to new products or novel technological advancements in the production process. Food neophobia appears to be a primary factor worsening consumers’ perceptions for UFs, as it does with several other innovative food products, from insect-based food [100] to cultured meat [101]. Studies have observed that food neophobia, as well as food technology neophobia, in a UF context, can be connected with factors that accentuate this negative consumer predisposition, especially related to production processes [94] or low education levels [95]. The extent to which the two types of neophobias affect consumers’ perception of upcycled products might also depend on the product itself. Coderoni and Perito [50,60] observed that both food and food technology neophobia have a relevant impact on the acceptance and purchase intention of waste-to-value foods with upcycled ingredients. However, the relevance of the two may differ in different contexts. For example, a study by Tsimitri et al. [102] on the acceptance of whey-based yogurt observed that consumers who expressed a neophobic attitude represented only a relatively small part of the sample. Similarly, Cattaneo et al. [95] observed, in a study in Italy, that most of the consumers were neophilic instead of neophobic when it came to UFs. In a similar fashion, risk aversion was described [94,98] to affect the perceived usefulness of technology behind UFs, with risk-averse individuals having lower WTP and intentions to purchase them compared to risk-taking individuals.

### 3.4. Consumer Perceptions

As depicted in Figure 5, consumer characteristics may play a crucial role in determining how product cues are perceived by consumers. Indeed, as theorized by the Total Food Quality model, consumers’ perceptions of product cues, rather than product cues per se, are the direct precursors to purchasing intentions.

Almost all the reviewed studies show that consumers have positive perceptions about the environmental benefits of UFs, which are one of the main drivers of acceptance for these foods [49,52,54,90]. These perceptions about environmental benefits may be related to different specific aspects, such as a reduction in food waste [62,66,85] or perceptions of environmentally friendly production practices and packaging [10].

UFs therefore tend to be perceived as more sustainable than their conventional counterparts [50,54], a perception that can be further enhanced by transparency, clarity, and accuracy in communicating their benefits [83].

It should be noted, however, that the association of UF with waste does not stimulate only positive responses due to perceived environmental benefits related to waste reduction. In some cases, this food-waste association might prompt suspicion and worries, leading some consumers to refrain from accepting this type of food [59].

While the perception of environmental benefits seems to be an established result of the literature on UF acceptance, more heterogeneous results are observed with respect to perceptions about health-related benefits. Specifically, some authors observed positive perceptions of consumers about health-related benefits of UFs [50,57,60,89,90]. In some cases, these perceptions were connected to a higher nutritional value of UFs [65] or to the presence of specific ingredients [78]. This was the case, for example, of the fiber content in a dairy-based flan dessert [90], or of high-protein, low-fat, and low-calorie characteristics [10]. However, in other cases, perceptions of UFs as healthier than their conventional counterparts appear in the absence of consumer knowledge about the product’s composition [22,52], suggesting that a halo effect may be at play.

Conversely, health-related benefits can also be negatively perceived in relation to UFs [94], stimulating negative effects, especially in risk-averse consumers [94,98].

Intrinsic and extrinsic product cues can also lead to the formation of specific perceptions in the fields of taste and overall quality. Often, consumers perceive upcycled products as of an overall higher quality than conventional ones [64,66], while in some cases, they even develop higher expectations in terms of taste [66], with positive reflections on purchase intentions [96].

Finally, the evaluation of price attributes hinges on how the price itself is perceived by consumers. Indeed, the higher price of upcycled products makes these products perceived as more expensive [83], potentially hindering their acceptance by price-sensitive consumers [64]. However, if consumers are made aware of the additional costs required to produce these products, their perceptions change, potentially changing to considering the higher price a fair one [83].

### 3.5. Sensory Characteristics

The right side of the horizontal dimension of the model addresses the after-purchase quality experience, which is only achieved after buying or consuming a particular food product. According to Grunert et al. [38], this experience stems from technical specifications, which determines the sensory characteristics of food products and affects consumers’ responses (Figure 5).

Among the experienced sensory attributes, taste plays a highly relevant role, and many studies highlighted that tasting UFs positively affects consumer responses. Mehta et al. [86] found that attitudes and intentions of consumers improved for ice cream made with upcycled ingredients after they tasted it. Goodman-Smith et al. [54] observed that the taste of upcycled craft beer in New Zealand turned out to be a major factor in consumer acceptability. Similarly, tasting bread enriched with wine by-products led to higher acceptability among Italian consumers [57], and comparable results were obtained by de Visser-Amundson et al. [103] in their study on potato-based upcycled sandwiches in the Netherlands. Investigating the role of taste in more depth, Lin et al. [77] observed that it is possible to increase the acceptability of UFs by acting on specific taste components (e.g., sweetness). However, a few cases were also observed where UFs did not perform better in terms of taste than their conventional counterparts. Teslic et al. [104], for example, found no relevant difference in consumer taste evaluation of upcycled pastries in comparison to a conventional counterpart. In other cases, UFs performed worse than their counterparts, as in the case of the lower taste experience for a grass protein-based cocoa drink [105] and flatbread made with tomato by-products [79].

Along with taste, other sensory attributes have been evaluated in the literature, with heterogeneous results. Talens et al. [68] observed that upcycled beef and lamb meat obtained positive scores for several sensory parameters, including appearance, aroma, and texture. Similar results were obtained by Tarjuelo [80] on lamb meat burgers for appearance, while no relevant difference from the conventional product was observed for texture. In the cereal and baked-goods sector, the aroma of cookies made with hazelnut skin led to higher purchase intentions in an Italian study [73], and the superior odor and aroma of a cherry pomace-filled pastry were highlighted in a Serbian sample of respondents [104]. Conversely, cereal bars with brewers’ spent grains, in the study of Stelick et al. [78], scored lower than their conventional counterparts for aroma, as well as for appearance and texture. Similar results for texture were observed by Man Le [106] for upcycled pasta, while no difference in product appearance emerged for upcycled pastries with sour cherry pomace filling [104].

The heterogeneity in the results suggests that the effect of upcycling on sensory parameters may be highly context-dependent, being affected by multiple elements, such as the main product and the characteristics of the upcycled ingredients, as well as the dose at which upcycled ingredients are used [79,106]. While these results hardly allow a generalization of the sensory effects of upcycling, what emerges is the opportunity to use upcycled ingredients to purposefully modify specific product sensory characteristics [75] to improve consumer acceptability.

## 4. Discussion

Our first research question inquired about consumer response toward UFs. Generally, consumer studies observe positive responses, portraying an optimistic scenario for the development of this food sector. However, some considerations are necessary, especially when examining how the share of studies with positive results varies according to different indicators used to measure consumer responses. Specifically, while most studies found positive results when investigating general attitudes and perceptions, the share of null and negative results increases as we move toward WTB or WTP. Essentially, the closer one moves toward actual consumption, the more heterogeneity is found in the literature; conversely, the further one moves away from consumption, the more studies agree on a positive consumer evaluation of UFs. This pattern aligns with a phenomenon commonly observed in several behavioral models of consumption, referred to as the ‘attitude-behavior’, the ‘attitude-intention’, or the ‘intention-behavior’ gap [72,107]. This gap is usually due to factors that prevent positive attitudes from translating into actual consumption behaviors [72,108,109]. Hence, the optimistic results emerging from our review should be considered with caution, as their actual translation into real-world impacts might not be straightforward. In this respect, while authors widely use hypothetical techniques to assess consumers’ responses to UFs, it seems particularly necessary for research to provide studies based on more realistic scenarios, where respondents are asked to make real purchases (e.g., experimental auctions). Incentive-compatible studies are, in fact, absent, at least among the studies reviewed in this work.

Improving the consumption of UF can be considered a valuable objective in light of sustainability considerations and food waste reduction. The studies we reviewed identify several levers to expand UF consumption. Specifically, referring to the theoretical model we adopted, the most promising paths to stimulate consumption of UFs are extrinsic product cues and consumer characteristics.

The provision of extrinsic product cues might, in fact, be useful to leverage specific consumer attitudes that are already present in the market or that might appear (or be stimulated) in the future. However, different types of cues exist that might perform different roles. For the purpose of discussion, we inductively classified product cues in three operational categories: “UF-specific cues”, “Easy access cues”, and “Accompanying cues” (see Table A3 in Appendix A for a classification of the studies investigating the three operational extrinsic cues categories). We define “UF-specific cues” as cues that can be used only in UFs due to their intrinsic link with the upcycling process. Examples include labels, certifications, and logos explicitly indicating the upcycled nature of the product, or claims highlighting positive impacts on food waste reduction. The potential of these cues is significant, as they allow clear differentiation of UFs on the market and thus enable the development of ad hoc marketing strategies. However, their effectiveness inevitably relies on consumer knowledge of what UFs are, which is still quite low in many contexts.

A second category of cues emerging from the literature is “Easy access cues”, which contain the cues that, because of upcycling, are relatively easy to attach to the product. This category includes many cues connected to sustainability and environmental preservation that communicate the ‘green nature’ of UFs. These cues appear particularly promising, as they align well with the attitudes of potential UF consumers, who often exhibit high environmental concerns and ecological purchase behaviors. Unlike “UF-specific cues”, the potential benefits of “Easy-access cues” are not exclusive to upcycled products. However, the valorization of these cues appears less dependent on the knowledge of UFs, as they are possibly appreciated by a larger segment of environmentalist consumers.

The last category of cues studied in the literature can be considered “Accompanying cues”. This category includes cues not specifically related to UFs but communicating specific characteristics of particular products (e.g., organic, healthiness, ingredients, and intrinsic attributes). As they affect consumers’ purchasing decisions, their role might be (as in any other food category) to segment the market to reach specific consumer targets. In this sense, they can play a key strategic role for UF producers, as they allow intra-category differentiation.

While the provision of cues can contribute to increasing consumer response toward UFs, consumer characteristics are another relevant factor. The literature highlights the great importance of individual environmental concerns as a driver of the propensity to consume UFs. This evidence suggests that, especially at the business level, devising sustainability-centered marketing and communication strategies might have great potential in fostering UF consumption. Such strategies would, in fact, allow companies to target environmental consumers and reap the benefits of their increased interest in UFs. From a public perspective, all efforts aimed at increasing environmental awareness possibly contribute to the increase in UF acceptance, despite increasing environmental consciousness being usually considered a long process [110].

Despite the potential positive role of environmental awareness, on a wider market level, a relevant barrier to the spread of UFs appears to be consumer knowledge and familiarity with these products. It follows that any action aimed at improving consumer knowledge of UFs has a significant potential for boosting UF consumption. In this respect, public interventions (e.g., through public campaigns) aimed at increasing the knowledge of these foods might be, in the short term, more effective in promoting UF consumption than those aimed at improving environmental awareness. Beyond this direct effect, knowledge of UFs and their processes can also attenuate the negative effects of other factors, such as food neophobia, risk aversion, or price sensitivity.

On the other hand, the role of private producers is twofold. Usual marketing tools (e.g., advertisement, package information) can be used to directly improve the knowledge of UFs to pursue their own marketing objectives. In addition, the placement of UF products on the market can stimulate consumer familiarity with them. This can potentially initiate a virtuous cycle, where increased UF knowledge favors higher UF acceptance, generating higher UF demand, which in turn stimulates a wider presence of UFs on the market, leading to a further increase in consumer familiarity with UFs.

Lastly, it is necessary to stress the role played by experienced quality. Despite the relatively scarce investigations on this topic in the reviewed literature, most studies agree on the necessity of a positive sensory evaluation of UFs for consumers to ultimately accept them. As such, it seems important that upcycling processes, even when they add value through extrinsic attributes (e.g., waste reduction claims, sustainability labels), do not undermine the sensory profile of foods. However, relevant opportunities emerge in connection with sensory evaluation. Specifically, several studies highlighted that upcycled ingredients can be used to confer desirable sensory characteristics to food products, thus improving consumer response.

## 5. Conclusions

Driven by increased attention to environmental sustainability, the spread of circular economy concepts, and advancing technological possibilities, the literature on UFs is growing rapidly, with consumer-related studies forming an integral part of this body of research.

While UF products are still in their introductory phase in many markets, promising avenues seem to be opening for them. Consumers appear interested and open to purchasing and consuming these products. They consider UFs sustainable, appreciate their quality, and are generally willing to buy them, although these positive impressions do not fully translate into a willingness to pay premium prices.

As expected, the sustainable/environmental motive is the leading factor driving consumer intentions toward these products. This suggests that consumers with strong environmental concerns will likely be the primary target to address for marketing these products. Consequently, emphasizing their environmental benefits appears to be a promising communication and marketing strategy to encourage their consumption.

Additionally, developing appropriate tools, such as certifications or voluntary labels, to communicate the upcycled nature shows potential, as such cues, as well as the intrinsic nature of UFs, are appreciated by specific segments of consumers. Therefore, the development of this type of instrument might be of interest for both private industries, which can add a tool to their marketing toolbox to target specific consumer groups, as well as for public institutions. The latter can, in fact, consider creating such kinds of tools to convey information to consumers interested in the consumption of UFs, basically solving possible information asymmetry issues.

However, a significant threat to the expansion of the UF sector is the limited knowledge about these products among consumers. This lack of awareness risks undermining the possibility that UFs will be evaluated more favorably than their standard counterparts. Low knowledge can impede the formation of consumer willingness to buy or pay price premiums. In some cases, it may even reinforce the expression of negative individual attitudes, such as food neophobia or risk aversion.

Nevertheless, the potential impact of communication and information actions is highly relevant. Most studies highlight that knowledge of UFs, along with individual environmental concerns, is among the main drivers of positive consumer responses. Based on this, one can expect, from both private and public perspectives (assuming UF consumption produces societal benefits), for the return from information and education actions to be substantial.

Considering the growth of the sector in recent years, we acknowledge that our contribution presents a static picture that will need to be expanded and refined in the future. While our innovative contribution was to summarize research discoveries on consumers’ responses to UFs using a consolidated theoretical lens, some limitations of our work can be addressed in the future. Among these, conducting a formal quality appraisal of the reviewed studies would make the results more robust, while expanding the research by relying on multiple databases, widening the search string, and including non-English material would widen the scope of the inquiry. In addition, our synthesis of the reviewed literature is based on a counting approach, which was used to detect the direction of consumer responses and of the effects of different factors on those responses. Further research can thus delve deeper into the investigation of the size of these effects, possibly also adopting meta-analysis approaches. Finally, as research on the topic is growing, future reviews can consider focusing on specific outcomes and research designs to investigate, in more detail, selected issues related to the consumer evaluation of UFs.

## Figures and Tables

**Table 1:** Number of studies analyzing different types of consumer responses towards UFs (percentages in parentheses).

Parameter/Response	Total Articles ^1^	Total Studies ^1^	Positive Results	Negative Results	Null Results
General perceptions	9 (16.66%)	12 (14.46%)	11 (91.67%)	1 (8.33%)	0 (0.0%)
Consumer acceptability	10 (18.51%)	15 (18.07%)	12 (80.0%)	2 (13.33%)	1 (7.1%)
Purchase intention/ Willingness to buy	22 (40.74%)	31 (37.35%)	24 (77.42%)	7 (22.58%)	0 (0.0%)
Willingness to pay	10 (18.51%)	12 (14.46%)	8 (66.67%)	4 (33.33%)	0 (0.0%)
Sensory evaluation	13 (24.07%)	13 (15.66%)	9 (69.23%)	4 (30.77%)	0 (0.0%)
Total	54 ^2^ (100%)	83 (100%)	64 (77.11%)	18 (21.69%)	1 (1.20%)

Note: By-column shares are reported for the *Total* columns, while by-row shares are reported for the other three columns (*Positive, Negative, Null*). ^1^ The differences between the number of articles and the number of studies are due to the presence of articles containing multiple distinct studies. This also implies that an article can study multiple different responses, causing the sum of the articles in the first column to be different from the total number of articles. ^2^ The sum across categories is 64 because there are some papers in which two or more than two different types of responses are studied.

## Data Availability

The original contributions presented in the study are included in the article, further inquiries can be directed to the corresponding author.

## Appendix A

**Table A1:** Coding table for mapping extracted variables into the four classes used in the synthesis of results.

Product Cues ^1^	Consumer Characteristics	Consumer Perceptions of UFs	Sensory Characteristics
Price (cost) UF labels, logos, and certifications (extrinsic) Information on upcycling processes (extrinsic) Waste reduction information (extrinsic) Environmental claims and certifications (extrinsic) Sustainability claims and certifications (extrinsic) Health claims (extrinsic) Nutritional claims/information (extrinsic) Food safety information (extrinsic) Organic certification (extrinsic) Indication of origin (extrinsic) Information on ingredients (extrinsic) Brand/Producer name (extrinsic) Color (intrinsic) Presence of damages (intrinsic) Texture (intrinsic) Overall visual appearance (intrinsic)	Familiarity with UFs Knowledge (subjective) of UFs Environmental consciousness Health consciousness Price consciousness Traditionalist attitude Self-reward attitude Social desirability/Social status attitude Food neophobia Food technology neophobia Risk aversion attitude Gender Level of education Age	Environmental benefits Health related benefits Higher nutritional value Higher overall quality Better taste expectations Food-waste negative association More expensive than conventional food	Taste Aroma Texture Odor Overall appearance Overall liking

^1^ In parentheses, we report the type of cue (cost, extrinsic, intrinsic) according to the revised TFQ model classification.

**Table A2:** Socio-demographic variables.

Variable	Response	Study	Operationalization	Effect (Direction)
Gender	General perceptions	[55]	Binary	Females have more favorable attitudes than males
		[84]	Binary	
		[95]	Binary	No gender differences
	Acceptability	[56]	Binary	No gender differences
		[88]	Binary	
	Purchase intention	[94]	Binary	Females have higher purchase intention
		[60]	Binary	No gender differences
		[66]	Binary	
	WTP	[87]	Binary	No gender differences
Age	General perceptions	[55]	Categorical	Young consumers display more favorable attitudes
		[84]	Categorical	
		[10]	Categorical	Middle-aged consumers display more favorable attitudes
		[95]	Categorical	No age differences
	Acceptability	[56]	Continuous	No age differences
		[88]	Continuous	
	Purchase intention	[66]	Categorical	Young consumers have the highest purchase intention; old consumers have the lowest
		[96]	Categorical	Old consumers have the highest purchase intention; young consumers have the lowest
		[94]	Categorical	No age differences
	WTP	[87]	Categorical	Young consumers display a higher WTP
Education	General perceptions	[55]	Binary (graduate: Y/N)	Highly educated consumers display more favorable attitudes
		[84]	Binary (graduate: Y/N)	No education differences
	Acceptability	[56]	Categorical	No education differences
		[88]	Categorical	
	Purchase intention	[60]	Binary (graduate: Y/N)	Consumers with higher education have higher purchase intention
		[65]	Categorical	Consumers with lower education have higher purchase intention
		[66]	Categorical	No education differences

**Table A3:** Classification of extrinsic product cues potentially improving consumer response toward UFs in the three operational cue categories used in the discussion. Studies from which the positive role of the specific cue is derived are reported in parentheses.

UF-Specific Cues	Easy Access Cues	Accompanying Cues
UF labels, logos, certifications and claims [26,49,54,59,60,64,65,83,95] Information on food waste reduction [55,66,70,82,86,92,95,105] Information on upcycling processes [66,87] Information on efficient use of resources [55,84]	Environmental claims (generic) [10,22,50,52,53,54,55,57,60,61,62,82,86,87,94,98,99,102] Sustainability claims [35,54,69,78,88,90] Sustainability certifications [82] Innovative product claims [55]	Nutritional claims [50,53,57,62,65,68,69,78,95,99] Health claims [22,50,52,57,60,61,87,89,102] Origin indication [50,53,56,65,85] Ingredients information [68,73,83,85,90] Organic certification [50,56,65] Claims on economic benefits [61,86,95] Food safety information [10,53,89] Brand/Producer name [26,50,105] Quality certifications [80] Naturalness claims [22]
